# N6-Methyladenosine Methylation Regulator RBM15 is a Potential Prognostic Biomarker and Promotes Cell Proliferation in Pancreatic Adenocarcinoma

**DOI:** 10.3389/fmolb.2022.842833

**Published:** 2022-02-09

**Authors:** Zhiying Zhao, Qiang Ju, Jing Ji, Yutong Li, Yanjie Zhao

**Affiliations:** ^1^ School of Public Health, Qingdao University, Qingdao, China; ^2^ Department of Blood Transfusion, The Affiliated Hospital of Qingdao University, Qingdao, China

**Keywords:** RBM15, genomic alteration, prognosis, immunity, proliferation

## Abstract

RNA binding motif protein 15 (RBM15) is a key regulatory factor involved in N6-methyladenosine (m6A) methylation. It has been reported that RBM15 plays an important role in the progress of laryngeal squamous cell carcinoma (LSCC), promoting LSCC migration and invasion. However, the role of RBM15 in human different cancers remains unknown. This study aims to analyze the prognostic value of RBM15, and to demonstrate the correlation between RBM15 expression and tumor immunity, as well as to provide clues for further mechanism research. The results showed that RBM15 was mutated or copy number varied in 25 types of cancer. RBM15 mRNA was abnormally up-regulated across various cancers. Survival analysis suggested high expression of RBM15 was associated with poor prognosis in many cancer types. Among these, it affected patients’ overall survival (OS) in 10 cancer types, disease-free interval (DFI) in 8 cancer types, progression-free interval (PFI) in 12 cancer types and disease-specific survival (DSS) in 7 cancer types. Importantly, in pancreatic adenocarcinoma (PAAD), overexpression of RBM15 is associated with patients’ OS, DFI, PFI, or DSS. In addition, RBM15 expression was positively correlated with immune infiltrating cells in kidney renal clear cell carcinoma (KIRC), brain lower grade glioma (LGG), and PAAD. Moreover, RBM15 expression showed a strong correlation with immune checkpoint markers in PAAD. Cell counting kit-8 (CCK-8) assay showed that knockdown of RBM15 significantly inhibited the proliferation of pancreatic cancer cells. PPI analysis showed USP10, USP24, SMG1, NRAS were closely connected with RBM15 alterations. Gene Ontology (GO) and Kyoto Encyclopedia of Genes and Genomes (KEGG) analysis showed that many biological processes (BP), cellular components (CC), molecular functions (MF), cancer related pathways including “sister chromatid cohesion”, “peptidyl-serine phosphorylation”, “cell division”, “nucleoplasm”, “nucleus”, “protein binding”, “protein serine/threonine kinase activity”, “T cell receptor signaling pathway”, “Cell cycle” were regulated by RBM15 alterations. Taken together, pan-cancer analysis of RBM15 suggested it may be served as a prognostic biomarker and immunotherapeutic target for PAAD.

## Introduction

N6-methyladenosine (m6A) methylation is the most abundant and common mRNA modification in higher eukaryotic cells (Qin et al., 2020; Zhang et al., 2020). RNA N6-methyladenosine is closely associated with cancer development and progression (Sun et al., 2019; Wang et al., 2020a). RBM15, as an m6A methyltransferase, is a key regulator in RNA m6A methylation (Jiang et al., 2021). It is involved in transcriptional inhibition mediated by XIST RNA (Patil et al., 2016; Brockdorff et al., 2020). In addition, it also functions as an mRNA export factor, stimulating export and expression of RTE-containing mRNAs (Lindtner et al., 2006; Zolotukhin et al., 2009). Study has shown that RBM15 promoted the progression of laryngeal squamous cell carcinoma (LSCC) by mediating the m6A modification of TMBIM6 mRNA (Wang et al., 2021). Although the role of RBM15 in LSCC is well understood, it’s functions in human pan-cancer remains largely unknown.

Recently, the research on tumor microenvironment (TME) has been developing. TME is a complex system formed by the interaction of tumor cells with surrounding tissues and immune cells (Anderson and Simon, 2020). The presence of TME regulated tumor cell proliferation, migration and immune escape, affecting tumorigenesis and progression (Wu et al., 2019; Hessmann et al., 2020). Tumor-associated macrophages (TAM) is one of the most common immune cells in the TME. TAM is involved in tumor progression (Li et al., 2020a; Lecoultre et al., 2020). Study has showed that in breast, ovarian and prostate cancers, increased amounts of TAM were positively correlated with tumor growth, invasive metastatic processes and poor treatment efficiency (Larionova et al., 2020). Thus, targeted TAM is a new strategy for cancer treatment (von Itzstein et al., 2020; Pathria et al., 2019; Cheng et al., 2021). In addition, immune escape is a major adverse event in cancer treatment (Yamamoto et al., 2020). Study has shown that activation of multiple immune escape mechanisms can drive the progression of lung cancer precancerous lesions to invasive lung cancer (Anichini et al., 2020). Therefore, the study of the role of tumor immune microenvironment in tumor progression is of great clinical value. However, the role of RBM15 in tumor immunity has not been elucidated.

In this study, we found that RBM15 is mutated and copy number varied in 25 cancer types. RBM15 is abnormally up-regulated and significantly associated with poor prognosis and immune infiltration in several cancer types, particularly in PAAD. Knockdown of RBM15 expression significantly inhibited proliferation of PAAD cell lines. In addition, functional enrichment analyses were performed using RBM15 alteration related genes. Our study sheds light on a possible role of RBM15 in different cancers, suggesting that RBM15 may be a potential prognostic and immunological biomarker in PAAD.

## Materials and Methods

### Pan-Cancer Analysis of Genomic Alterations of RBM15

cBioPortal database (http://www.cbioportal.org/, v3.6.20) was used to analyze the genomic profile of RBM15 in human cancers, including mutations and copy number variations. Four mismatch repair (MMR) genes’ (MLH1, MSH2, MSH6, PMS2) mutation levels were downloaded through TCGA database (http://tcga-data.nci.nih.gov, v23.0). Pearson correlation analysis was used to evaluate the relationship between RBM15 expression and MMR gene mutation levels.

### Pan-Cancer Analysis of Transcriptional Levels of RBM15

The difference in RBM15 transcriptional levels between cancers and normal tissues was analyzed by combining the data for normal tissues from Genotype Tissue Expression (GTEx) database (https://gtexportal.org/, v8) with the data from The Cancer Genome Atlas (TCGA). RNA sequencing data of 33 cancer types included, ACC: adrenocortical carcinoma, BLCA: bladder urothelial carcinoma, BRCA: breast invasive carcinoma, CESC: cervical squamous cell carcinoma, CHOL: cholangiocarcinoma, COAD: colon adenocarcinoma, DLBC: lymphoid neoplasm diffuse large B-cell lymphoma, ESCA: esophageal carcinoma, GBM: glioblastoma multiforme, LGG: brain lower grade glioma, HNSC: head and neck squamous cell carcinoma, KICH: kidney chromophobe, KIRC: kidney renal clear cell carcinoma, KIRP: kidney renal papillary cell carcinoma, LAML: acute myeloid leukemia, LIHC: liver hepatocellular carcinoma, LUAD: lung adenocarcinoma, LUSC: lung squamous cell carcinoma, MESO: mesothelioma, OV: ovarian serous cystadenocarcinoma, PAAD: pancreatic adenocarcinoma, PCPG: pheochromocytoma and paraganglioma, PRAD: prostate adenocarcinoma, READ: rectum adenocarcinoma, SARC: sarcoma, SKCM: skin cutaneous melanoma, STAD: stomach adenocarcinoma, TGCT: testicular germ cell tumors, THCA: thyroid carcinoma, THYM: thymoma, UCEC: uterine corpus endometrial carcinoma, UCS: uterine carcinosarcoma, UVM: uveal melanoma were obtained from TCGA database. All data were normalized as previously described (Ju et al., 2020a; Ju et al., 2020b).

### Prognosis Analysis

The relationship between RBM15 expression and OS, DFI, PFI and DSS in 33 types of cancer were analyzed by forest plots and Kaplan-Meier curves. The hazard ratio (HR) and log-rank *p* values were acquired by univariate survival analysis.

### Correlation Between RBM15 Expression and Immune Characteristics

Immune infiltrating cell scores for 33 cancer types were downloaded from the Tumor Immune Evaluation Resource (TIMER) database (https://cistrome.shinyapps.io/timer/, v2.0). The correlation between RBM15 expression and six immune infiltrating cells (B cell, CD4 + T cell, CD8 + T cell, neutrophil cell, macrophage cell, and dendritic cell) were analyzed by Spearman correlation. In addition, we selected 47 common immune checkpoint markers for our study. Pearson correlation analysis was used to assess the correlation between RBM15 expression and immune checkpoint markers level.

### Cell Culture

The pancreatic cancer cell lines SW1990 and PANC-1 were obtained from Laboratory of Department of Health Toxicology, Qingdao University (Qingdao, China). All cells were cultured in DMEM medium (BasalMedia, Shanghai) containing 10% fetal bovine serum (FBS; BioInd, Israel) and 1% penicillin–streptomycin with 5% CO2 at 37°C in a humidified incubator.

### siRNA Transfection

Pancreatic cancer cells were seeded on 12-well plates 1 day before the experiment. siRNA was transfected (called si-RBM15) and negative control (called NC) when the density confluence reached 60–80%. siRNA for transfection was purchased from GenePharma (China Shanghai). Transfection was performed using Lipofectamine RNAiMAX reagent (Invitrogen, INC, United States) according to manufacturer’s instructions.

### RNA Extraction, Reverse Transcription and Real-Time Quantitative PCR (qRT-PCR)

Total RNA was extracted from SW1990 and PANC-1 cells using Invitrogen TRIzol reagent (Invitrogen, United States). Total RNA was reversed into cDNA using the PrimeScript™ RT kit (Takara, Dalian, China) for qRT-PCR detection of the RBM15 gene, according to the manufacturer’s protocol. qRT-PCR analysis was performed using the SYBR Premix Ex Tap Kit (Takara, Dalian, China) based on the LightCycler^®^ 480 system (Roche, Basel, Switzerland). In this study, relative mRNA expression was normalized to GAPDH, both based on 2−ΔΔCq method. All experiments were conducted at least 3 times.

### Cell Proliferation Assay

For cell proliferation assay, the transfected cells were seeded into 96-well plates at a density of 3000 cells per well. At 24, 48, 72,96 and 120h after seeding, cell viability was measured by the CCK-8 system (Dojindo, Japan) according to the manufacturer’s instructions. Briefly, each well was added with 10 μl CCK- 8 solution and the plate was incubated at 37°C with 5% CO2 for 2 h in dark. The absorbance was measured at 450 nm with a microplate reader (Thermo Fisher, China, Shanghai).

### Functional and Pathway Enrichment Analysis

First, the correlation between co-expressed genes and RBM15 expression which in 175 TCGA samples with mutations or CNA variation was analyzed by Spearman correlation analysis. *p* < 0.05, R ≥ 0.4 and R ≤ -0.4 were considered to be significant. The association of co-expressed genes was analyzed by PPI network through the STRING database (https://www.string-db.org, v11.0). GO and KEGG analysis were performed by DAVID database (https://david.ncifcrf.gov, v6.8) as previously described (Ju et al., 2020c; Zhao et al., 2021). GO analysis focuses on biological process (BP), cellular component (CC) and molecular function (MF). KEGG was used to analyze the pathways associated with 82 co-expressed genes associated with RBM15 mutations.

### Statistical Analysis

The *t* test was used to evaluate the expression difference of RBM15 in tumor tissues and normal tissues. Univariate survival analysis and Kaplan-Meier survival analysis were used to analyze the correlation between RBM15 expression and patients’ survival. For correlation analysis, *p* < 0.05 and R > 0.20 were considered to be relevant. *p* < 0.05 were considered to be significance for all statistical analysis.

## Results

### Genomic Alterations of RBM15 in Human Pan-Cancer

**FIGURE 1:**
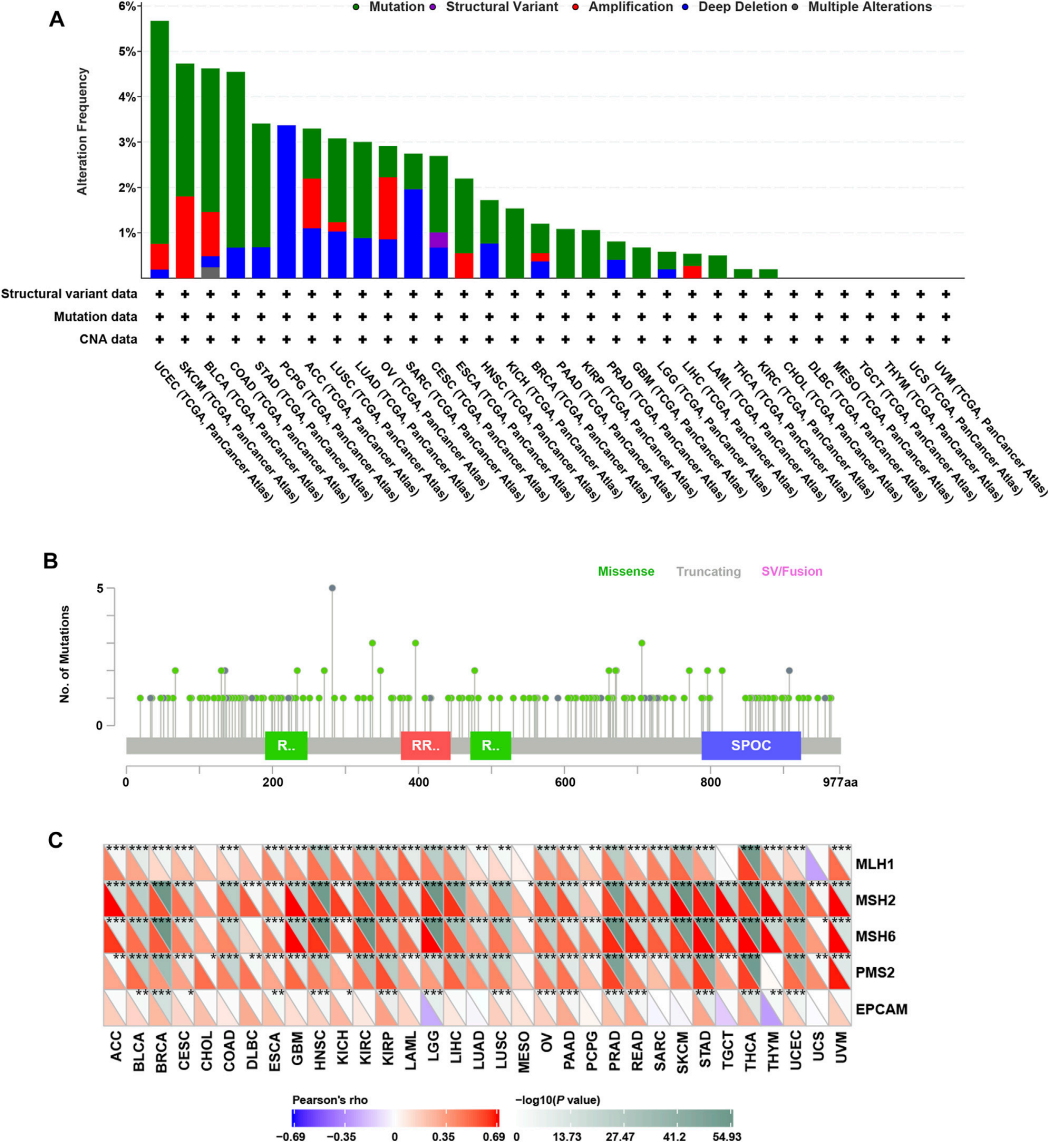
Genomic alterations of RBM15 in human pan-cancer. **(A)** The alteration frequency of RBM15 across tumors. **(B)** The types and distributions of RBM15 mutations. X-axis, amino acid; Y-axis, numbers of RBM15 mutations; green/red box, RNA recognition motif (190-248aa, 376-444aa, 471-527aa); blue box, SPOC domain (788-924aa). **(C)** Correlation of RBM15 expression with four MMR genes mutation.

Genomic alterations including mutations and copy number variations often occur in cancer (Futreal et al., 2004; Van Bockstal et al., 2020). Firstly, we analyzed genomic alterations of RBM15 gene in 32 cancers by cBioPortal database. Interestingly, as shown in Figure 1A, we found that RBM15 was mutated or copy number varied in 25 cancer types, including UCEC, SKCM, BLCA, COAD, STAD, PCPG, ACC, LUSC, LUAD, OV, SARC, CESC, ESCA, HNSC, KICH, BRCA, PAAD, KIRP, PRAD, GBM, LGG, LIHC, LAML, THCA, KIRC. Among these, there were 18 types of cancer including UCEC, SKCM, BLCA, COAD, STAD, LUSC, LUAD, CESC, ESCA, KICH, BRCA, PAAD, KIRP, GBM, LGG, LAML, THCA, KIRC, which’s genomic alteration were mainly mutation. However, copy number deletion is the main type of genomic alteration in PCPG and SARC. Additionally, in ACC, OV, HNSC, PRAD and LIHC, mutation and copy number variation coexisted. Furthermore, 175 RBM15 mutations were identified across various cancers, 146 (83.4%) were missense, 28 (16%) were truncating and 1 (0.6%) was SV/Fusion (Figure 1B, Supplementary Table S1).

DNA mismatch repair (MMR) maintains genomic stability (Ijsselsteijn et al., 2020). Mutations in MMR gene might cause defective mismatch repair, leading to genomic alterations of some genes (Peña-Diaz and Rasmussen, 2016). Next, we investigated the correlation of four MMR genes’ mutation and RBM15. Results showed RBM15 expression was significantly correlated with the mutation levels of MMR genes in most tumor types (Figure 1C). All these results indicate RBM15 exists genomic alterations in many cancers.

### RBM15 mRNA Is Aberrantly Up-Regulated in Human Pan-Cancer

**FIGURE 2:**
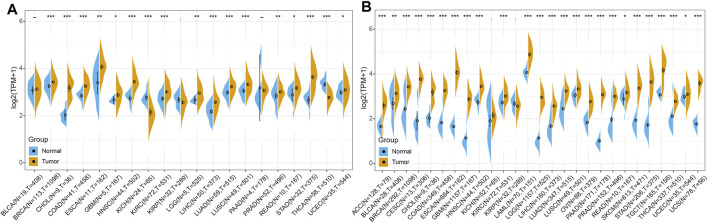
RBM15 is aberrantly expressed in human pan-cancer. **(A)** Abnormal expression of RBM15 in tumor and normal tissues from TCGA database. **(B)** RBM15 is aberrantly overexpressed in 25 tumor types from TCGA and GTEx database. *p < 0.05, **p < 0.01, ***p < 0.001.

In order to elucidate the difference of RBM15 expression between tumor and normal, the expression data of RBM15 in 20 types of cancers including BLCA (N = 19, T = 408), BRCA (N = 113, T = 1098), CHOL (N = 9, T = 36), COAD (N = 41, T = 458), ESCA (N = 11, T = 162), GBM (N = 5, T = 167), HNSC (N = 44, T = 502), KICH (N = 24, T = 65), KIRC (N = 72, T = 531), KIRP (N = 32, T = 289), LGG (N = 5, T = 525), LIHC (N = 50, T = 373), LUAD (N = 59, T = 515), LUSC (N = 49, T = 501), PAAD (N = 4, T = 178), PRAD (N = 52, T = 496), READ (N = 10, T = 167), STAD (N = 32, T = 375), THCA (N = 58, T = 510), UCEC (N = 35, T = 544) were analyzed using the TCGA database. As shown in Figure 2A, RBM15 transcriptional levels were significantly higher in BRCA, CHOL, COAD, ESCA, GBM, HNSC, KIRC, LGG, LIHC, LUAD, LUSC, PRAD, READ, STAD and UCEC than in normal tissues. Considering that the sample size of normal tissues in the TCGA database was relatively small, we then integrated the GTEx data and the TCGA data to analyze the expression differences of RBM15 in 27 tumor types including ACC (N = 128, T = 79), BLCA (N = 28, T = 408), BRCA (N = 292, T = 1098), CESC (N = 13, T = 306), CHOL (N = 9, T = 36), COAD (N = 349, T = 458), ESCA (N = 664, T = 162), GBM (N = 1157, T = 167), HNSC (N = 44, T = 502), KICH (N = 52, T = 65), KIRC (N = 72, T = 531), KIRP (N = 32, T = 289), LAML (N = 70, T = 151), LGG (N = 1157, T = 525), LIHC (N = 160, T = 373), LUAD (N = 347, T = 515), LUSC (N = 49, T = 501), OV (N = 88, T = 379), PAAD (N = 171, T = 178), PRAD (N = 152, T = 496), READ (N = 10, T = 167), SKCM (N = 813, T = 471), STAD (N = 206, T = 375), TGCT (N = 165, T = 156), THCA (N = 337, T = 510), UCEC (N = 35, T = 544), UCS (N = 78, T = 56). Results showed that RBM15 mRNA levels in 25 tumor types including ACC, BLCA, BRCA, CESC, CHOL, COAD, ESCA, GBM, HNSC, KIRC, LAML, LGG, LIHC, LUAD, LUSC, OV, PAAD, PRAD, READ, SKCM, STAD, TGCT, THCA, UCEC and UCS were significantly higher than in normal tissues (Figure 2B). These results suggest that RBM15 is abnormally up-regulated in various cancers.

### Prognostic Value Analysis of RBM15 in Human Pan-Cancer

**FIGURE 3:**
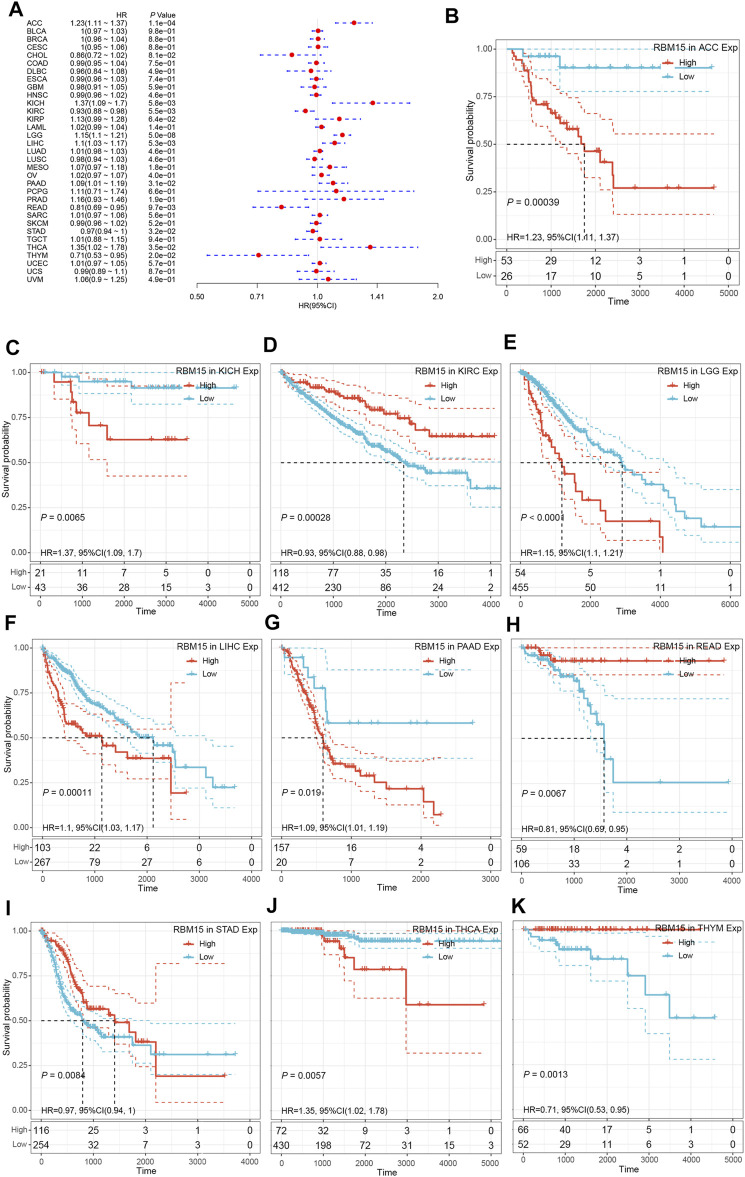
RBM15 expression is correlated with patient’ OS. **(A)** Forest plots of HR for RBM15 expression in 33 tumor types. **(B–K)** Kaplan-Meier curves of significant correlation between RBM15 expression levels and patient’ OS in ACC, KICH, KIRC, LGG, LIHC, PAAD, READ, STAD, THCA and THYM.

Next, we investigated whether abnormal expression of RBM15 affect patients’ prognosis. By univariate survival analysis, we found that RBM15 expression was associated with patients’ OS in 10 cancer types, including ACC, KICH, KIRC, LGG, LIHC, PAAD, READ, STAD, THCA and THYM (Figure 3A).In addition, Kaplan-Meier OS curves showed that increased RBM15 expression was correlated with poor prognosis in 6 cancer types including ACC (*p* = 0.00039, HR = 1.23), KICH (*p* = 0.0065, HR = 1.37), LGG (*p* < 0.0001, HR = 1.15), LIHC (*p* = 0.00011, HR = 1.1), PAAD (*p* = 0.019, HR = 1.09) and THCA (*p* = 0.0057, HR = 1.35). However, KIRC (*p* = 0.00028, HR = 0.93), READ (*p* = 0.0067, HR = 0.81), STAD (*p* = 0.0084, HR = 0.97) and THYM (*p* = 0.0013, HR = 0.71) were exceptions where RBM15 overexpression indicated a better prognosis (Figures 3B–K).

**FIGURE 4:**
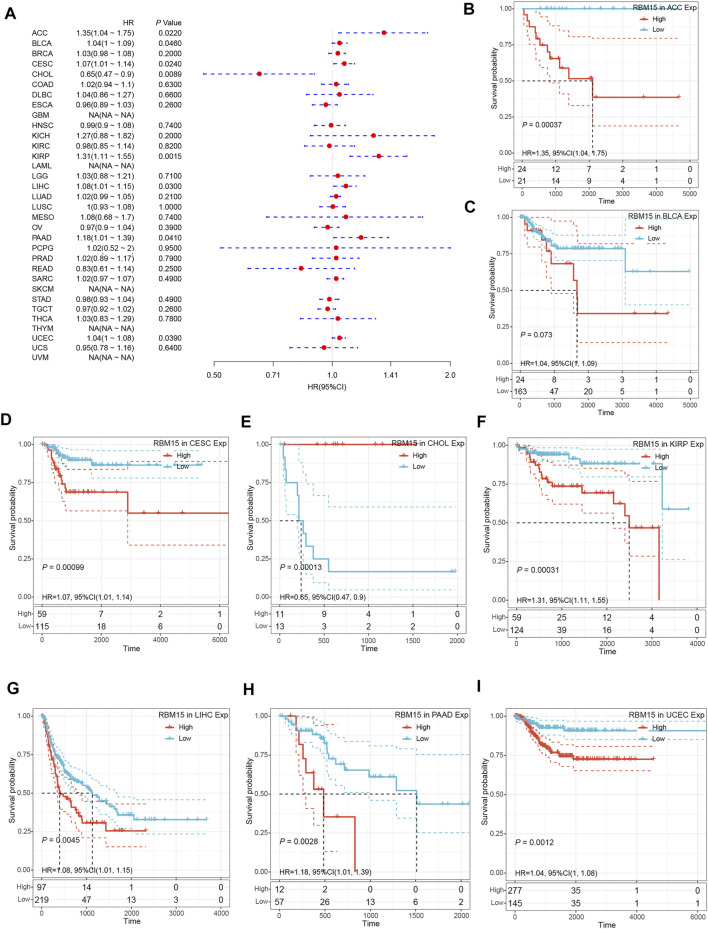
RBM15 expression is correlated with patient’ DFI. **(A)** Forest plots of HR for RBM15 expression in 33 tumor types. **(B–I)** Kaplan-Meier curves of significant correlation between RBM15 expression levels and patient’ DFI in ACC, BLCA, CESC, CHOL, KIRP, LIHC, PAAD and UCEC.

**FIGURE 5:**
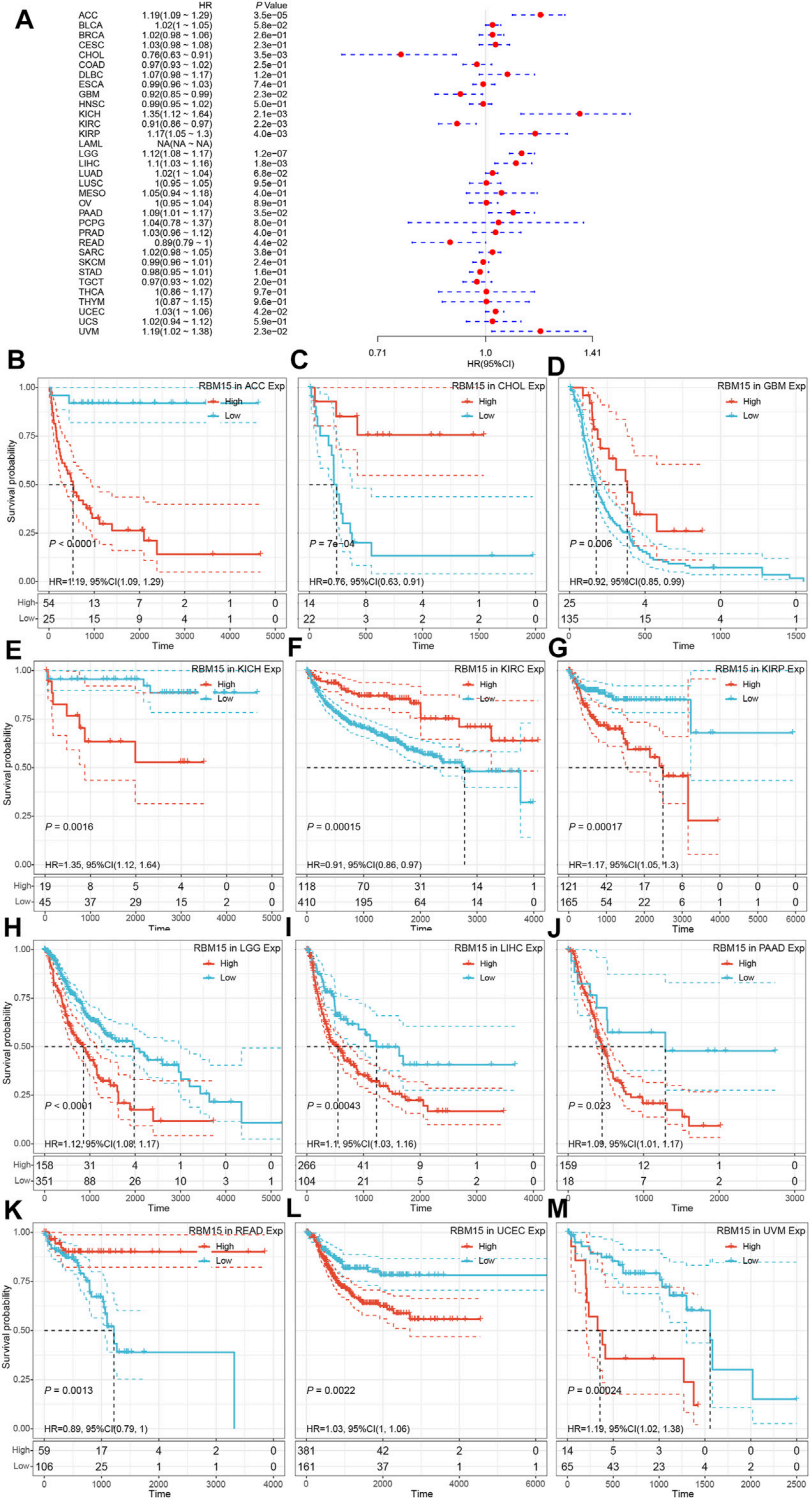
RBM15 expression is correlated with patient’ PFI. **(A)** Forest plots of HR for RBM15 expression in 33 tumor types. **(B–M)** Kaplan-Meier curves of significant correlation between RBM15 expression levels and patient’ PFI in ACC, CHOL, GBM, KICH, KIRC, KIRP, LGG, LIHC, PAAD, READ, UCEC and UVM.

**FIGURE 6:**
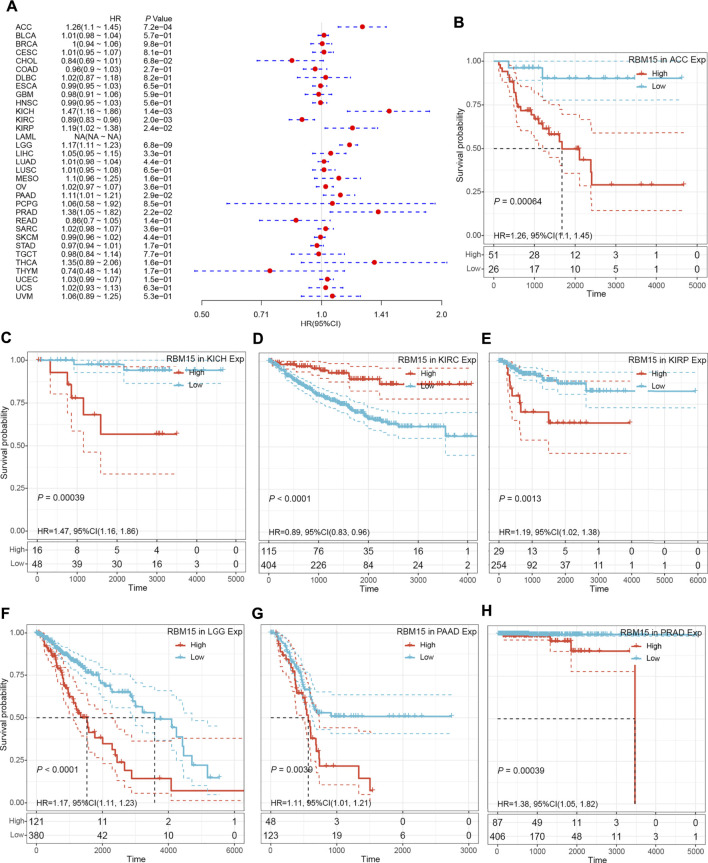
RBM15 expression were related to patient’ DSS. **(A)** Forest plots of HR for RBM15 expression in 33 tumor types. **(B–H)** Kaplan-Meier curves of significant correlation between RBM15 expression levels and patient’ DSS in ACC, KICH, KIRC KIRP, LGG, PAAD and PRAD.

In addition, we examined the effect of RBM15 expression on patient’ DFI. As shown in Figure 4A, RBM15 expression was associated with patients’ DFI in 8 cancer types, including ACC, BLCA, CESC, CHOL, KIRP, LIHC, PAAD and UCEC. Kaplan-Meier DFI curves showed that increased RBM15 expression was associated with poor prognosis in ACC (*p* = 0.00037, HR = 1.35), BLCA (*p* = 0.073, HR = 1.04), CESC (*p* = 0.00099, HR = 1.07), KIRP (*p* = 0.00031, HR = 1.31), LIHC (*p* = 0.0045, HR = 1.08), PAAD (*p* = 0.0028, HR = 1.18), UCEC (*p* = 0.0012, HR = 1.04), and reversely in CHOL (*p* = 0.00013, HR = 0.65) (Figures 4B–I). Moreover, we also analyzed the correlation between RBM15 expression and patient’ PFI, and found that abnormal RBM15 expression affected patients’ PFI in 12 cancer types, including ACC, CHOL, GBM, KICH, KIRC, KIRP, LGG, LIHC, PAAD, READ, UCEC and UVM (Figure 5A). Kaplan-Meier PFI curves showed that increased RBM15 expression was associated with poor prognosis in ACC (*p* < 0.0001, HR = 1.19), KICH (*p* = 0.0016, HR = 1.35), KIRP (*p* = 0.00017, HR = 1.17), LGG (*p* < 0.0001, HR = 1.12), LIHC (*p* = 0.00043, HR = 1.1), RAAD (*p* = 0.023, HR = 1.09), UCEC (*p* = 0.0022, HR = 1.03) and UVM (*p* = 0.00024, HR = 1.19). However, in CHOL (*P* = 7e-04, HR = 0.76), GBM (*p* = 0.006, HR = 0.92), KIRC (*p* = 0.00015, HR = 0.91) and READ (*p* = 0.0013, HR = 0.89), high expression of RBM15 showed a good prognosis (Figures 5B–M). Additionally, we also evaluate effect of RBM15 overexpression on patients’ DSS. Forest plot showed that high expression of RBM15 was associated with DSS in patients with 7 types of cancer, including ACC, KICH, KIRC KIRP, LGG, PAAD and PRAD (Figure 6A). Kaplan-Meier DSS survival analysis revealed that high RBM15 expression induced short DSS in ACC (*p* = 0.00064, HR = 1.26), KICH (*p* = 0.00039, HR = 1.47), KIRP (*p* = 0.0013, HR = 1.19), LGG (*p* < 0.0001, HR = 1.17), PAAD (*p* = 0.0039, HR = 1.11) and PRAD (*p* = 0.00039, HR = 1.38), but inversely in KIRC (*p* < 0.0001, HR = 0.89) (Figures 6B–H). These results suggest that RBM15 expression is strongly associated with patients’ prognosis in many cancers, especially in PAAD, whether OS, DFI, PFI or DSS.

### High Expression of RBM15 Is Related to Immunity

**FIGURE 7:**
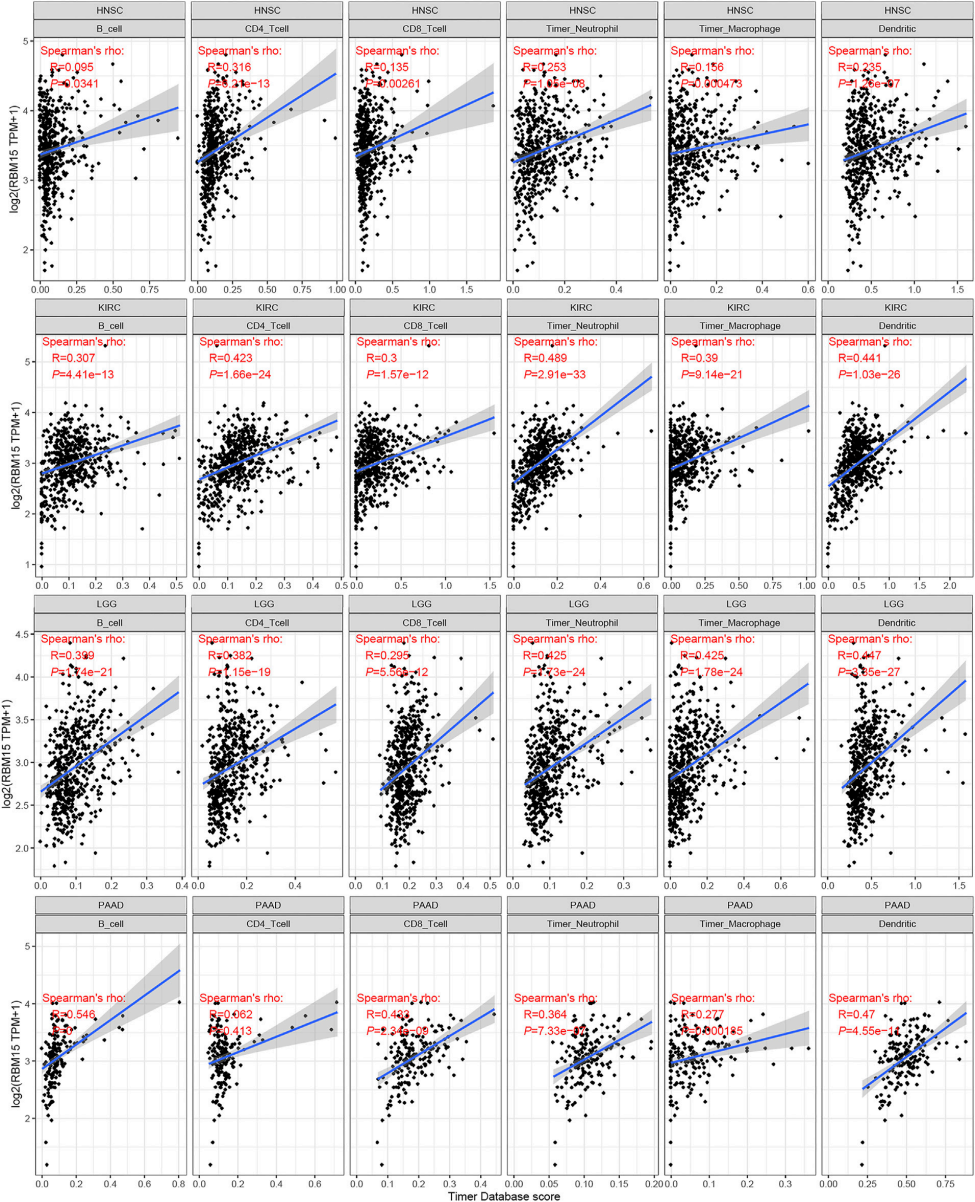
Correlation of RBM15 expression with immune infiltration level of 6 types of immune cells in HNSC, KIRC, LGG, PAAD.

The immune cells in TME can affect patients’ survival (Qin et al., 2021). To explore the mechanism of RBM15 affecting patients’ prognosis, the correlation between RBM15 expression and immune infiltration in pan-cancer was further investigated. First, we analyzed the scores of 6 types of immune cells (B cell, CD4 + T cell, CD8 + T cell, neutrophil cell, macrophage cell, and dendritic cell) from 33 cancer types through the TIMER database. Results showed that the expression of RBM15 was significantly correlated with 6 or 5 types of immune cells in HNSC, KIRC, LGG. PAAD (Figure 7).

**FIGURE 8:**
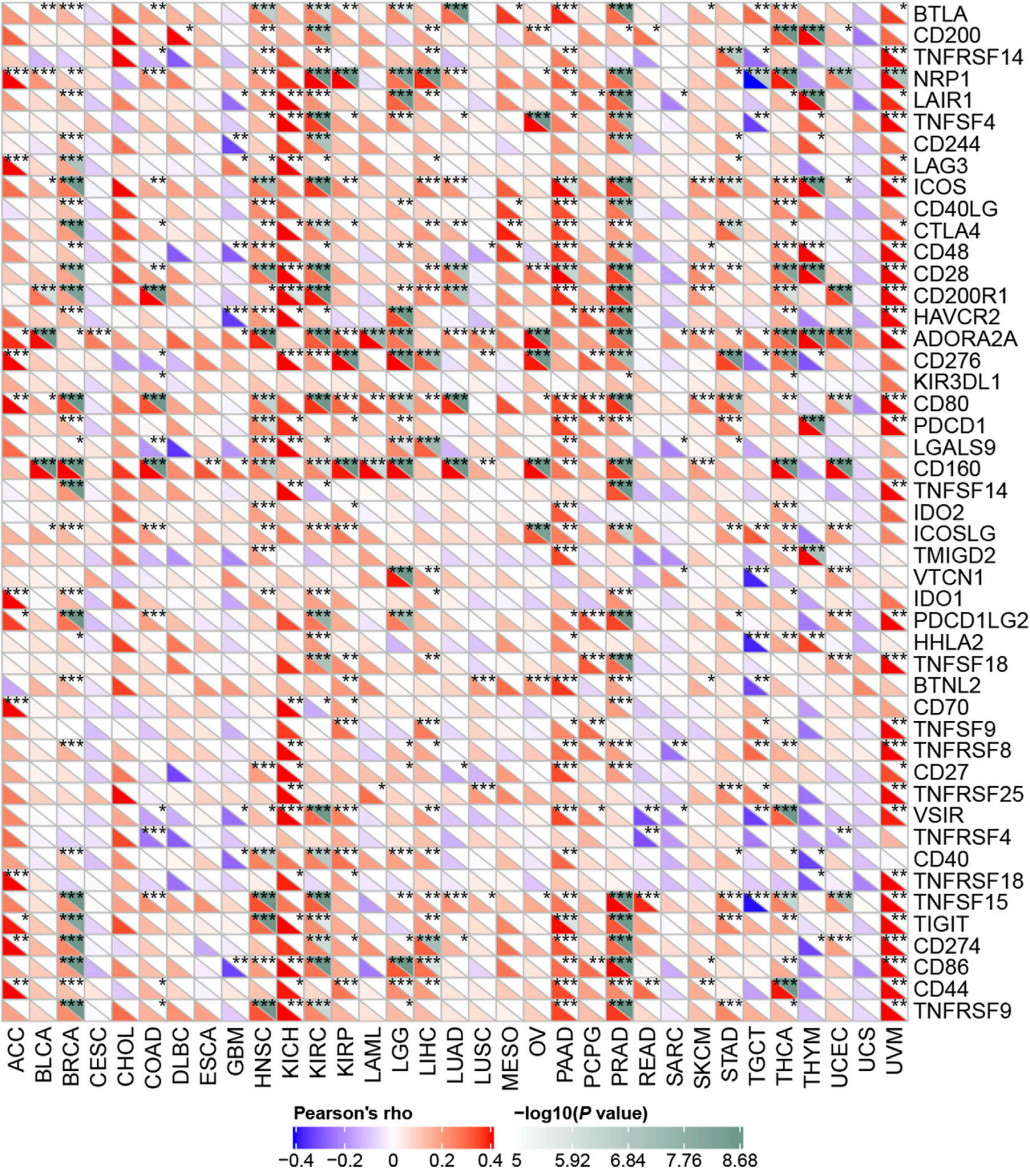
Correlation analysis of RBM15 expression levels with 47 immune checkpoints in human pan-cancer. *p < 0.05, **p < 0.01, ***p < 0.001.

Next, we explored how RBM15 affected immune cells infiltration. The correlation between RBM15 expression and immune checkpoint gene expression was investigated. Interestingly, we found that in BRCA, HNSC, KIRC, PAAD, PRAD and UVM, RBM15 expression was significantly associated with more than 30 immune checkpoint markers, such as TNFRSF9, CD86, TIGIT, CD80, etc. (Figure 8). Taken together, these results suggest that in KIRC and PAAD, RBM15 plays an important role in immunity.

### Knockdown of RBM15 Inhibited the Proliferation of Pancreatic Cancer Cells

**FIGURE 9:**
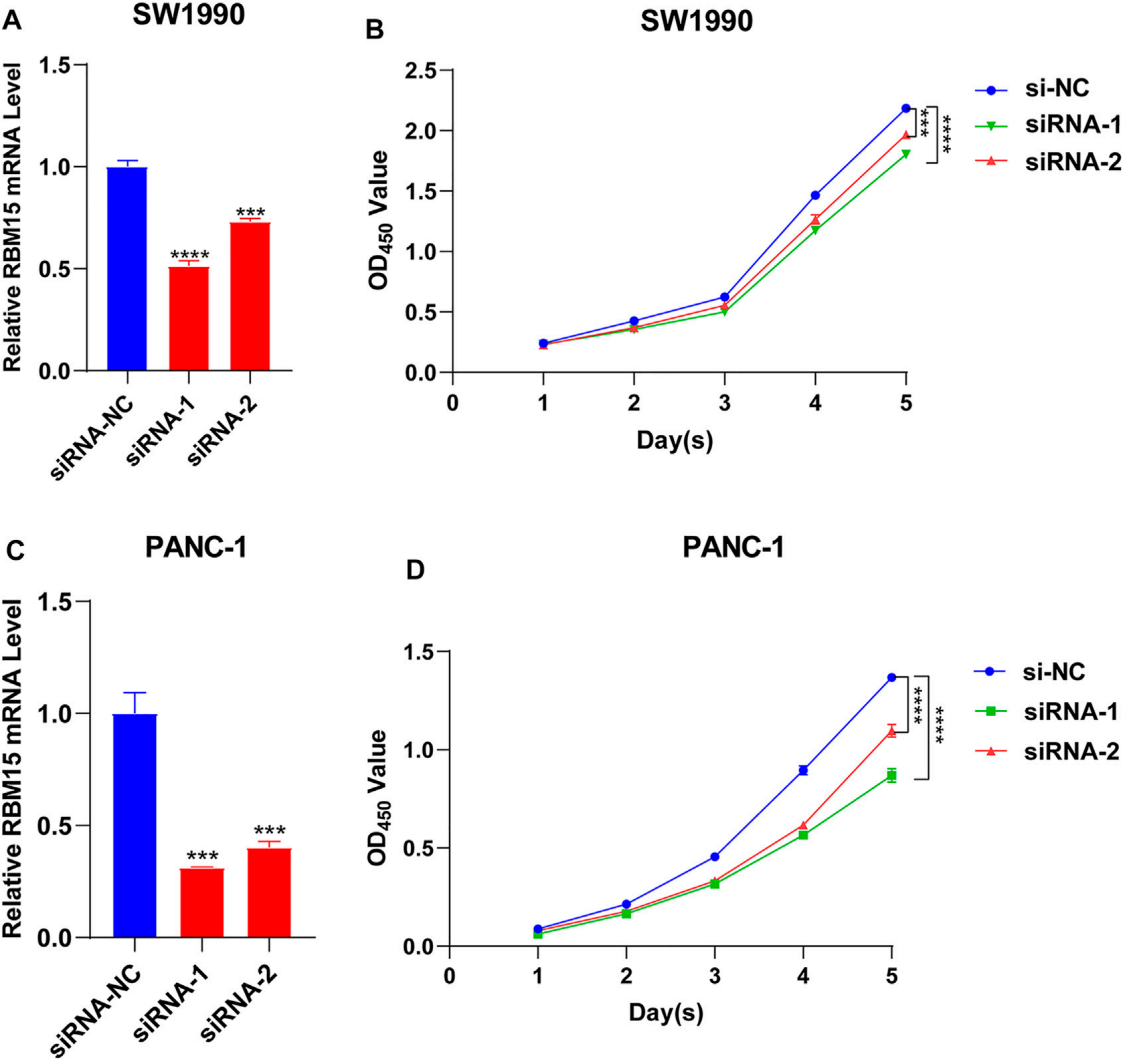
Knockdown of RBM15 inhibited proliferation of pancreatic cancer cells. **(A)** The knockdown efficiency of siRNA was detected by qRT-PCR in SW 1990. **(B)** CCK-8 showed that knockdown of RBM15 inhibited proliferation of SW 1990. **(C)** The knockdown efficiency of siRNA was detected by qRT-PCR in PANC-1. **(D)** CCK-8 showed that knockdown of RBM15 inhibited proliferation of PANC-1.

All of the above results indicated that RBM15 had genomic alterations and was up-regulated in PAAD. It may affect the survival of patients by regulating the immune microenvironment. Next, we detected the biological phenotype caused by RBM15 in PAAD cell lines. Firstly, we transfected siRNA of RBM15 in SW1990 and PANC-1 cells and found that RBM15 mRNA were significantly decreased in these cells (Figures 9A,C). CCK-8 assay results showed that knockdown of RBM15 significantly inhibited the proliferation of PAAD cells. (Figures 9B,D).

### Functional Enrichment Analysis of RBM15 and 82 Co-Expressed Genes in PAAD

**FIGURE 10:**
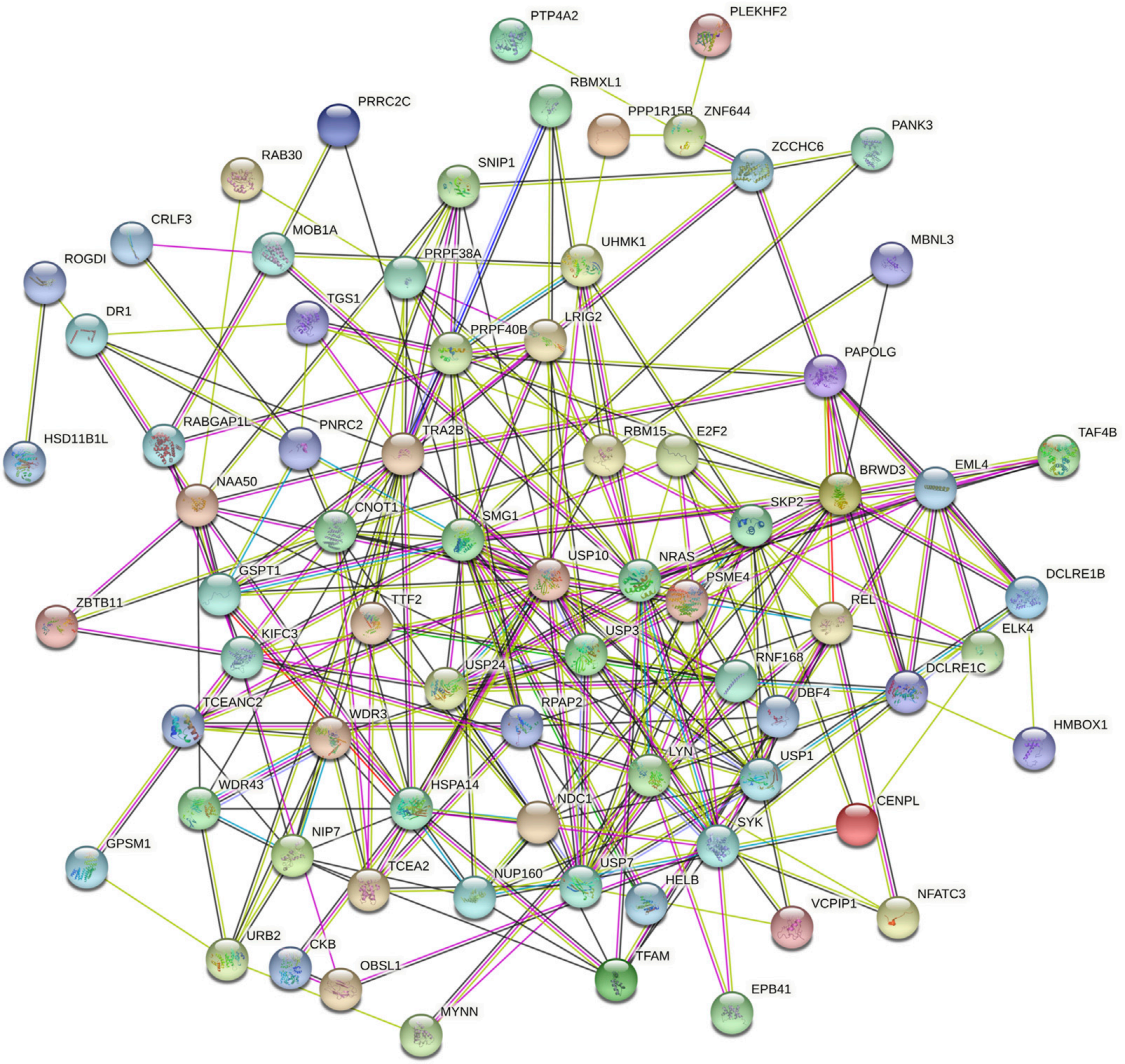
The PPI network of RBM15 and its 82 co-expression genes from STRING database.

**FIGURE 11:**
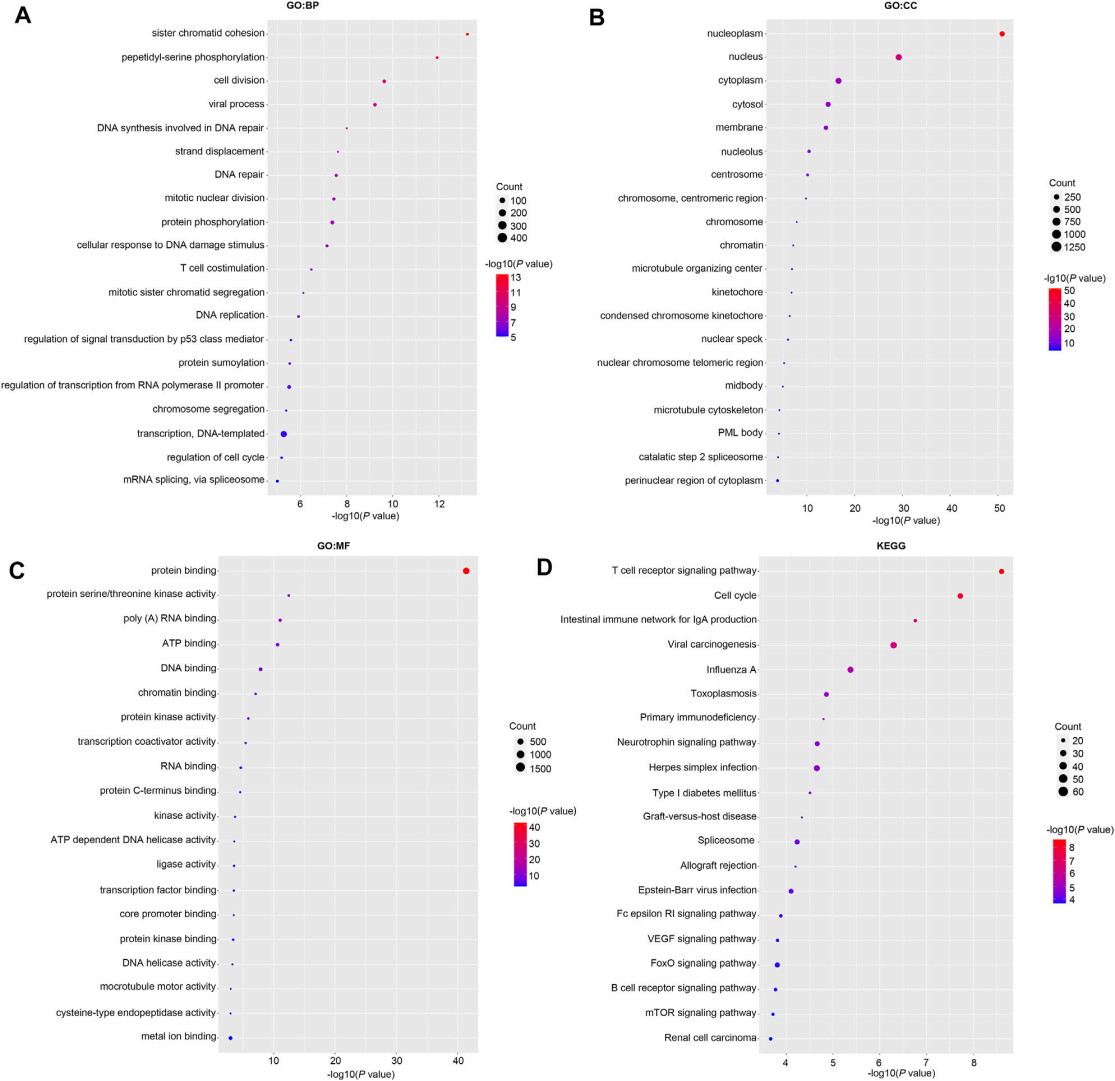
GO and KEGG analysis of 82 co-expression genes of RBM15 alterations in PAAD. **(A)** GO: BP. **(B)** GO: CC. **(C)** GO: MF. **(D)** KEGG.

Then we investigated the signal pathway RBM15 regulated in PAAD. By cBioPortal database, we obtained 82 co-expressed genes that were significantly associated with RBM15 alteration (Supplementary Table S2). Then PPI network was constructed through the STRING database. As shown in Figure 10, we obtained USP10, USP24, SMG1, NRAS were closely related to RBM15 alterations. In addition, GO analysis indicated these co-expressed genes were significantly enriched in many BP, CC, MF, including “sister chromatid cohesion”, “peptidyl-serine phosphorylation”, “cell division”, “nucleoplasm”, “nucleus”, “protein binding”, “protein serine/threonine kinase activity” (Figures 11A–C). The significant pathway enriched by KEGG included “T cell receptor signaling pathway”, “Cell cycle” (Figure 11D).

## Discussion

Pan-cancer analysis focuses on studying alterations in DNA, RNA and protein levels in different human tumor types to reveal genes associated with tumor development, which is important for cancer prevention and improving patient survival (Chen et al., 2021; Lu et al., 2021). As an epigenetic regulatory molecular, RNA N6-methyladenosine plays a crucial role in the development and progression of cancer (Wang et al., 2018a). m6A modification was reversibly regulated by “Writers”, “Erasers” and “Readers”. The methyltransferase complex, known as “Writers”, consists of METTL3, METTL14, METTL16, WTAP, KIAA1429, ZC3H13 and RBM15/15B (Shi et al., 2019). The demethylases are classified as “Erasers” and included FTO and ALKBH5 (Jia et al., 2011; Zheng et al., 2013). “Readers” are m6A binding proteins, including YTHDC1/2, YTHDF1/2/3, IGF2BP1/2/3, HNRNPG, HNRNPC, and HNRNPA2B1 (Bi et al., 2019). RBM15, as a “Writer” of m6A, has been shown to play an important role in the progression of LSCC in previous study (Wang et al., 2021). Although RBM15 has been extensively studied in LSCC, its roles in pan-cancer and whether it can be used as a biomarker remains unclear. In this study, we first analyzed genomic alterations of RBM15 in human pan-cancer and found that there were mutations or copy number variations in RBM15 genome. Additionally, RBM15 was abnormally high expressed in 25 cancers and correlated with patients’ poor prognosis and immunity in PAAD. GO and KEGG analysis showed RBM15 alteration was significantly associated with cancer and immune related signaling pathways, such as “cell division”, “T cell receptor signaling pathway” and “Cell cycle”. Taken together, these results suggest that RBM15 may be a potential and immunological biomarker.

Genomic instability is an important feature of cancer development (Ben-David et al., 2019). Genomic instability mainly consists of two forms: mutations and copy number variants (Negrini et al., 2010; Keefe, 2020). Study showed genetic mutations can drive tumor development (Martínez-Jiménez et al., 2020). Mutations in the human gene on chromosome 22 could cause bladder cancer (Zhang and Zhang, 2015). In addition, copy number variation inhibits or promotes cancer progression (Shao et al., 2019). Study has showed that RAS amplification should as a predictor of anti-EGFR therapy in metastatic colorectal cancer (Schrock et al., 2021). All of these indicate that there are mutations or copy number variations in genes related to the occurrence and development of cancer. In this study, we found that RBM15 is mutated or has copy number variation and significantly correlated with MMR gene mutations across many cancer types.

Previous study has shown that RBM15 could promote invasion and accelerate the malignant progression of LSCC (Wang et al., 2021). In addition, high expression of RBM15 affects the prognosis of HBV-associated hepatocellular carcinoma patients (Fang and Chen, 2020). The previous studies all suggest RBM15 may be abnormally expressed in cancers and affect cancer development and progression. In this study, we found that RBM15 mRNA levels were higher in 25 tumor types than in normal tissues. These results suggest that RBM15 may be an important cancer-related gene.

Studies have shown that abnormal gene expression is associated with poor prognosis of cancer (Liu et al., 2019; Song et al., 2020; Zhou et al., 2020; Han et al., 2021). In the current study, we analyzed the effect of abnormal RBM15 expression on patients’ survival. Results showed that high expression of RBM15 was associated with poor prognosis in several cancer types, particularly in PAAD. Regardless of OS, DFI, PFI or DSS, PAAD patients with high RBM15 expression had shorter survival times than PAAD patients with low RBM15 expression. These results suggest that RBM15 may be a potential prognostic marker for PAAD.

Tumor immune microenvironment plays a dual role in cancer: on the one hand, it acts as a cancer suppressor by destroying cancer cells or inhibiting their growth; on the other hand, it promotes tumor development by providing tumor cells with the suitable conditions for tumor growth (Schreiber et al., 2011). Tumor-infiltrating immune cells, as an important part of tumor immune microenvironment, includes B cells, T cells neutrophils, macrophages and dendritic cells (Wang et al., 2019). Studies have shown that regulatory B cells promote tumor progression by cross-regulating with tumor cells (Shang et al., 2020). B cells selectively promotes breast cancer lymph node metastasis through HSPA4-targeted IgG (Gu et al., 2019). IL35-producing B cells promote pancreatic cancer tumor development (Pylayeva-Gupta et al., 2016). Lactate regulates CD4^+^ T cell polarization and induces immunosuppression to promote prostate cancer progression through the TLR8/miR21 axis (Comito et al., 2019). Activated CD8 T cells stimulate the EMT process in breast epithelial tumor cells and increase the tumorigenic capacity of breast cancer cells (Reiman et al., 2010). Neutrophils play an important role in promoting liver metastasis for colon cancer (Mizuno et al., 2019). Macrophages can contribute to the malignant progression of cancer by promoting tumor cell invasion and suppressing anti-tumor immunity (Cassetta and Pollard, 2018). Dendritic cells in the tumor microenvironment support angiogenesis, block anti-tumor immune responses and stimulate cancer cell growth and spread (Ma et al., 2012). All these studies indicate that tumor infiltrating immune cells play an important role in the development of tumor. In this study, we found RBM15 expression was significantly associated with immune infiltrating cells including B cell, CD4 + T cell, CD8 + T cell, neutrophil cell, macrophage cell, and dendritic cell in KIRC, LGG and PAAD. Immune checkpoints are frequently upregulated in a variety of malignancies to facilitate tumor growth and are highly expressed in dysfunctional CD8+T cells (Toor et al., 2020). Furthermore, the correlation between RBM15 and immune checkpoint markers implies an important role of RBM15 in regulating tumor immunity, especially in PAAD. Next, we knocked down the expression of RBM15 mRNA in pancreatic cancer cell lines SW1990 and PANC-1, which significantly inhibited cell proliferation. These results further suggest an important role of RBM15 in tumor immunity and proliferation.

In this study, we screened 82 co-expressed genes associated with RBM15 alterations and performed PPI analysis. The results of PPI demonstrated significant co-expression of genes associated with RBM15 alterations, include USP10, USP24, SMG1 and NRAS. USP10 appears to be particularly important in the network. Previous studies showed USP10 inhibited tumor cell growth in wild-type P53 cells and as a modulator of the tumor suppressor P53 (Yuan et al., 2010). USP10 also promoted hepatocellular carcinoma proliferation by deubiquitylation and stabilization of YAP/TAZ (Zhu et al., 2020). USP10 inhibited lung tumorigenesis by activating the KLF4-TIMP3 pathway (Wang et al., 2020b). Moreover, studies have shown that USP24 promoted cancer malignant progression by stabilizing p300 and β-TrCP-induced IL-6 in the tumor microenvironment (Wang et al., 2018b). In addition, USP24 promoted lung cancer malignancy by stabilizing proteins containing bromodomains (Wang et al., 2020c). MiR-18a exerted its oncogenic effects by inhibiting SMG1 and activating the mTOR pathway in nasopharyngeal carcinoma (NPC) cells (Mai et al., 2019). NRAS mutations drive melanoma progression (Qian et al., 2020). These results strongly suggest that RBM15 alteration associated co-expressed genes are closely associated with the development of cancer.

The GO and KEGG pathway analysis showed RBM15 alteration was associated with immune and cancer related signaling pathways. GO analysis analyzed the BP, CC and MF parts. During the BP, CC and MF process, “sister chromatid cohesion”, “peptidyl-serine phosphorylation”, “cell division”, “nucleoplasm”, “nucleus” and “protein binding” were significantly enriched. In KEGG analysis, the most important signaling pathway was “T cell receptor signaling pathway”, which was closely related to cancer. Studies have showed that Chimeric antigen receptor T cells (CAR T) inhibited the malignant progression of glioblastoma, non-small cell lung cancer, and breast cancer (Li et al., 2020b; Qu et al., 2021; Toulouie et al., 2021). There are other pathways involved in cancer, such as the “cell cycle”. Cell cycle is closely related to the fate of cells (Dalton, 2015). Cell cycle dysregulation is the main factor leading to abnormal proliferation of tumor cells (Ahmad et al., 2020). EGR1 has been reported to promote gastric cancer cell cycle progression and tumorigenesis through the mediated linc01503 (Ma et al., 2021). Together, these results provide clues for further investigation of the mechanism of RBM15 in cancer.

## Conclusion

This study shows that RBM15 has mutations and copy number variations in human pan-cancer. High expression of RBM15 is associated with poor prognosis and tumor immunity in many cancers, especially in PAAD. These results suggest that in PAAD, RBM15 might be an immunotherapeutic target and a promising prognostic biomarker.

## Data Availability

The original contributions presented in the study are included in the article/Supplementary Material, further inquiries can be directed to the corresponding author.
